# Distinct metabolic network states manifest in the gene expression profiles of pediatric inflammatory bowel disease patients and controls

**DOI:** 10.1038/srep32584

**Published:** 2016-09-02

**Authors:** Carolin Knecht, Christoph Fretter, Philip Rosenstiel, Michael Krawczak, Marc-Thorsten Hütt

**Affiliations:** 1 Institute of Medical Informatics and Statistics, Christian-Albrechts University Kiel, Kiel, Germany; 2 Department of Life Sciences and Chemistry, Jacobs University, Bremen, Germany; 3 Institute of Clinical Molecular Biology, Center for Molecular Biosciences, Christian-Albrechts University Kiel, Kiel, Germany

## Abstract

Information on biological networks can greatly facilitate the function-orientated interpretation of high-throughput molecular data. Genome-wide metabolic network models of human cells, in particular, can be employed to contextualize gene expression profiles of patients with the goal of both, a better understanding of individual etiologies and an educated reclassification of (clinically defined) phenotypes. We analyzed publicly available expression profiles of intestinal tissues from treatment-naive pediatric inflammatory bowel disease (IBD) patients and age-matched control individuals, using a reaction-centric metabolic network derived from the Recon2 model. By way of defining a measure of ‘coherence’, we quantified how well individual patterns of expression changes matched the metabolic network. We observed a bimodal distribution of metabolic network coherence in both patients and controls, albeit at notably different mixture probabilities. Multidimensional scaling analysis revealed a bisectional pattern as well that overlapped widely with the metabolic network-based results. Expression differences driving the observed bimodality were related to cellular transport of thiamine and bile acid metabolism, thereby highlighting the crosstalk between metabolism and other vital pathways. We demonstrated how classical data mining and network analysis can jointly identify biologically meaningful patterns in gene expression data.

Over the past decade, the advent and further development of the high-throughput molecular techniques of genomics, proteomics and metabolomics have rendered possible the generation of rich molecular data sets at ever increasing speed. Due to the mere size and complexity of these data, however, both hypothesis-driven analyses and agnostic data mining exercises are usually hampered by serious multiple comparison problems. In consequence, molecular studies of human disease have rarely led to more than long lists of uninterpretable fold changes and p values, with little direct benefit to scientific scrutiny. Occasionally, selected experimental targets may also accrue from the expertise of individual research groups, but the evidence basis of such ‘good guesses’ is usually subjective or sparse, or both. Based upon previous experience in other areas of scientific research, it may thus be surmised that proper contextualization of molecular data by additional biological information would greatly facilitate their interpretation at different levels of cellular organization.

The term ‘network medicine’ has been coined to summarize attempts at gaining a systemic understanding of biological processes by mapping experimental data onto networks^1^. These networks serve as abstractions of the underlying biological processes and, in this way, render them more amenable to statistical and mathematical analysis. In fact, throughout the distinguished career of network-based science^2^, the question of how to use biological networks to interpret high-throughput molecular data has played an important role^3^,^4^. Yet, all strategies brought forth so far essentially follow the same principle: Data attributes are associated with vertices in a network of interest and are given statistical weight depending upon their bonding by network edges.

Crohn disease (CD) and ulcerative colitis (UC) are inflammatory bowel diseases (IBD), characterized by relapsing-remitting episodes of intestinal inflammation. Both entities provide prime examples of a complex disease that is caused by a poorly understood interplay between environmental and genetic risk factors. Usually, both diseases first arise between the 2^nd^ and 4^th^ decade of life and have a strong effect upon the quality of life of patients. More specifically, CD and UC are associated with pain and bloody diarrhea, have debilitating inflammatory extra-intestinal manifestations (e.g. arthritis, uveitis), and require strong and long-term immunosuppressive medication. Both diseases are associated with a Western lifestyle and have become dramatically more frequent in the second half of the 20^th^ century^5^. Genetic studies identified a wealth of replicated disease associations to over 160 genomic regions^6^, suggesting an important role of immune signaling, endoplasmic reticulum (ER) stress, autophagy and cytoskeletal organization in IBD etiology. Despite the large number of risk loci and the improved understanding of their functional role, however, the exact causes of IBD still remain to be elucidated. There is currently no cure for either CD or UC, and primary and secondary non-response to induction and maintenance therapy represent a major problem of IBD clinical care.

Unsupervised gene expression analysis of patient samples aims at a better understanding of those gene regulatory processes that are critical for disease etiology, progression and treatment response. However, despite several fruitful attempts to follow this paradigm in the case of IBD^7^,^8^,^9^,^10^, ways and means to infer different functional states of patient tissue from gene expression profiles, and to relate these states to the disease phenotype of interest, are still missing. Here, we follow an archetypical ‘network medicine’ approach to infer hitherto unrecognized patterns in gene expression data from IBD and control mucosal samples. We hypothesized that one or more deregulated states of a biological network may exist in the patients and that this variation can be identified from gene expression profiles taking the natural variation between patients properly into account.

Metabolic networks seem to suggest themselves as plausible candidates for network medicine in the IBD context because the human body makes many metabolic adjustments in response to, and in order to compensate for, inflammatory processes. The relevance of metabolic organization in IBD pathophysiology has been recognized early on^11^ but systematic studies of IBD-related metabolic gene activity are still lacking. Therefore, extracting effective metabolic networks from gene expression changes in IBD patients may be an ideal test case for such a systems-based approach and, at the same time, may reveal new hints at the biological mechanisms underlying the disease. Moreover, distinct metabolic states may be associated with differences in disease progression and may therefore point towards a meaningful stratification of patients with a view on treatment and surveillance. Finally, complementing networks with standard enrichment analysis may allow metabolism-related states to be linked to the utterance of other biological functions.

## Results and Discussion

**Figure 1:**
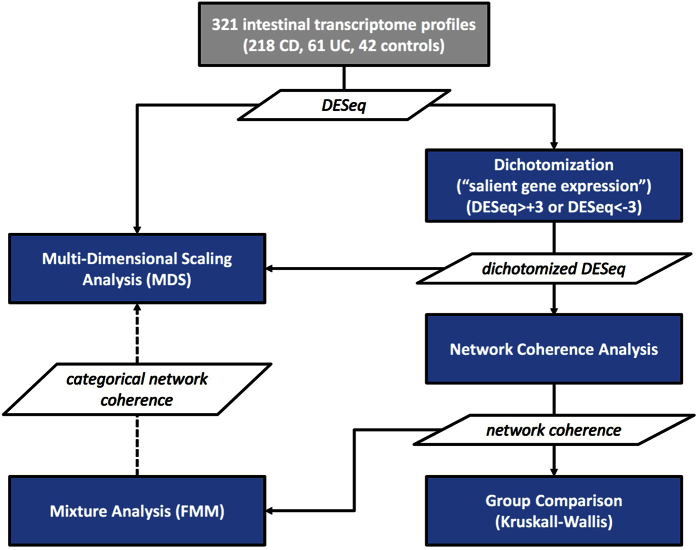
Flow chart depicting the different data processing and analysis steps of the study.

In the present study, we focused upon the utility of metabolic networks to contextualize molecular data. More specifically, we used the Recon2 metabolic model^12^ as a template to interpret publicly available gene expression profiles^13^ of intestinal tissue from control individuals and treatment-naive pediatric patients diagnosed with either Crohn disease (CD) or ulcerative colitis (UC). This age group may be rather untypical for IBD. However, we surmise that the analysis of pediatric patients may shed some extra light on the etiological link between gene expression and disease manifestation because, around the incidence peak of 20 to 40 years, this relationship may already be confounded to a considerable extend by past or present environmental influences. Our study involved multiple data processing and analysis steps (Fig. 1) that combine a metabolic network-based approach to data analysis with classical data mining, jointly facilitating a more function-orientated interpretation of the expression profiles.

### Quantification of metabolic coherence

The concept of metabolic network coherence employed here^14^,^15^ is based upon genome-wide metabolic networks that are subjected to flux-balance analysis (FBA), a variant of constraint-based modeling^16^. FBA starts from the solution space of a linear system, *N*∙*v* = 0, with stoichiometric matrix *N* and metabolic flux vector *v*. After the inclusion of necessary constraints (e.g., maximal nutrient uptake rates or reversibility of biochemical reactions), an objective function (e.g., biomass maximization) is defined and the optimal flux is found by linear programming^17^,^18^. FBA has been applied successfully in microbiology before, for example, to predict gene essentiality with high accuracy for *Escherichia coli*^19^ and *Saccharomyces cerevisiae*^20^. With the publication of the first metabolic models of human cells^21^,^22^ and their multiple refinements^12^,^23^, an application of the concept of metabolic network coherence in human medical research has become feasible. Our analysis strategy^15^ was first applied to gene expression profiles from patients with aldosterone-producing adenomas of the adrenal gland, where it revealed several distinct metabolism-related states in the data. Similar approaches combining flux prediction with gene expression profiling have been used, for example, to establish cell type-specific metabolic models^23^,^24^,^25^.

The metabolic network derived from the Recon2 model is a bipartite graph with metabolite nodes and reaction nodes. A projection of this bipartite graph onto the reaction nodes (i.e. the reaction-centric metabolic network) and the evaluation of the gene-reaction associations contained in Recon2 lead to a (gene-centric) metabolic network with vertices representing genes and edges representing paths of length 2 between the gene-associated reactions in the original bipartite graph. We analyzed effective metabolic networks that were obtained by mapping significantly altered gene expression levels onto the gene-centric metabolic network. Here, ‘significantly altered’ gene expression was defined by way of calling a gene ‘saliently expressed’ in a given profile when the normalized expression (DESeq; see below) value for that gene exceeded ± 3. Note that ± 3 is an appropriate threshold for z scores like the normalized DESeq values because ± 3 roughly demarcates the 1% quantile of the standard Gaussian distribution. The general principle of metabolic network coherence analysis is depicted in Supplementary Figure S1.

**Figure 2:**
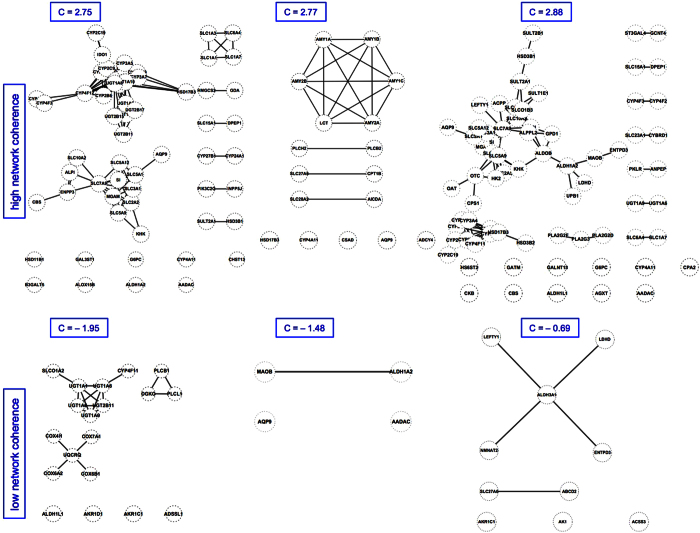
Examples of effective networks. Top row: effective networks of high metabolic coherence; bottom row: effective networks of low metabolic coherence. Standard gene names from the Recon2 metabolic model were used. For example, ALPL denotes the gene encoding alkaline phosphatase, liver/bone/kidney. C: metabolic network coherence.

A central problem of metabolic network coherence analysis in its original form^15^ has been the choice of an appropriate objective function and of suitable input to the metabolic system (i.e., a suitable cellular environment). We circumvented this problem by using a static network rather than a network comprising predicted active fluxes obtained via FBA. Statistically, the main effect of FBA in network coherence analysis is meaningful pruning of the original (usually dense) reaction-centric metabolic network. We achieved a similar effect by eliminating currency metabolites (ATP, H_2_O, etc.) from the bipartite metabolic network before projecting the set of reaction nodes onto the network (see Methods section for additional information). Examples of both high and low coherence effective networks generated in the course of our study are shown in Fig. 2.

Network analysis yielded a single global quantity per individual, called the ‘metabolic network coherence’ of the corresponding gene expression profile. Formal assessment by means of a Kruskall-Wallis test revealed a highly significant difference in metabolic network coherence between the three diagnostic groups (χ^2^ = 9.305, 2 d.f., p = 0.0095). The observed heterogeneity was entirely due to a lower level of coherence prevailing in the expression profiles of controls (median: −0.195) compared to CD (0.596) and UC (0.723) patients. No significant difference was observed between CD and UC (p > 0.2).

### Multi-modality of metabolic network coherence

**Figure 3:**
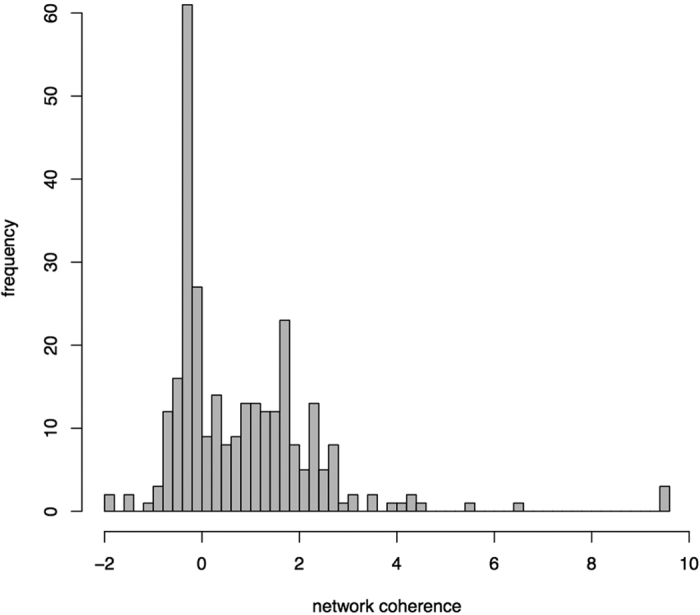
Distribution of metabolic network coherence in all intestinal samples.

**Figure 4:**
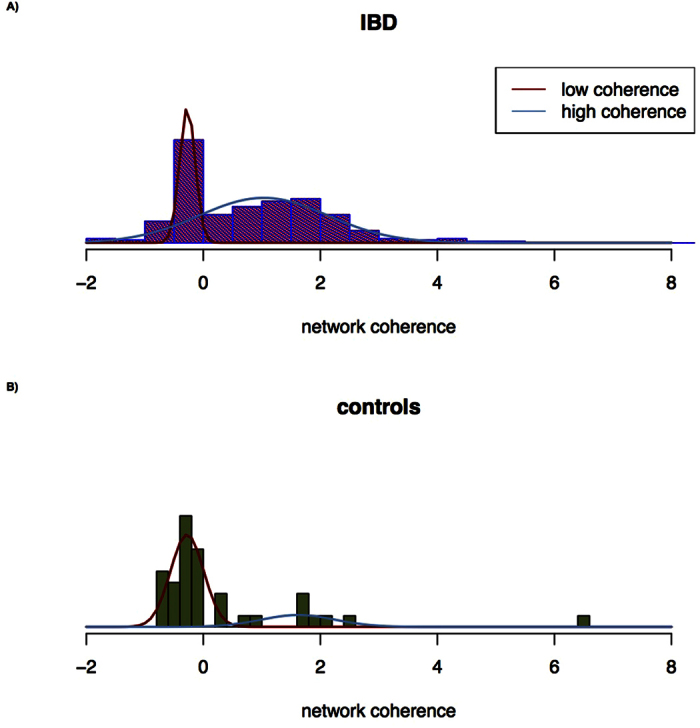
Distribution of metabolic network coherence in intestinal samples, stratified by IBD status.

Visual inspection further revealed that the distribution of metabolic coherence values was characterized by prominent multi-modality (Fig. 3). The significance and precise stochastic nature of this finding were formally evaluated by mixture analysis as implemented in SAS procedure FMM (version 9.5; SAS Institute Inc., Cary, NC, USA). Since FMM is unsuitable for the analysis of heavily skewed distributions, we applied a standardized extreme deviation criterion^26^,^27^ to define outliers as values more than 5.2 median absolute deviations away from the median (equivalent to a metabolic network coherence value > 3.578). Applying this threshold highlighted seven IBD samples and four control samples as outliers. Upon the exclusion of these values, use of a Bayes Information Criterion (BIC) yielded the best fit to the data for a mixture of two Gaussian distributions with mixing probabilities 0.267 (A) and 0.733 (B) (see Fig. 4A). Mean and variance were estimated as −0.272 and 0.017, respectively, for distribution A, and 1.029 and 1.206, respectively, for distribution B. Mixture analysis of individual patient subgroups yielded similar results for CD and UC, with nearly identical means but somewhat different variances (Supplementary Figure S2; Supplementary Table S1). Statistically significant substructure, as judged by a BIC, was also detected in the control profiles. Again, the best fit to the data was obtained with a mixture of two Gaussian distributions, and the respective mean and variance estimates were −0.278 and 0.080 for distribution A, and 1.618 and 0.403 for distribution B. Whilst these parameters were strikingly similar to those characterizing the metabolic network coherence distributions in patients, however, the mixing probabilities were reversed at 0.777 for distribution A, and 0.223 for distribution B (Fig. 4B).

High metabolic network coherence is obtained when expression level differences between different genes fit to the topology of the metabolic network, i.e. when expression levels tend to be more similar for genes that are connected in the network than would be expected by chance alone. This kind of coherence can be interpreted as meaning that the expression profile is partially ‘explicable’ by the network. For individuals with low metabolic network coherence, by contrast, other functional characteristics (beyond the metabolism-related state) would have to be invoked to ‘explain’ their gene expression profile.

The above results suggest that the intestinal gene expression profiles of children can be subdivided into two groups, one with metabolic network coherence of high average level and large variance, and one with notably lower average and smaller variance. These two subgroups are present at relative frequencies of approximately 1:3 in pediatric treatment-naive IBD patients, and 3:1 in same-aged controls, i.e. IBD is strongly associated with intestinal gene expression of high metabolic coherence. In principle, there are two basic explanations for this observation. Either high metabolic coherence or the biological causes thereof represent a risk factor for IBD at young age *per se*. In this case, our results potentially point towards novel disease mechanisms worth further exploration. Alternatively, the development or presence of pediatric IBD may cause a shift of gene expression from low to high coherence in some patients, but not in others. Even although our results would then lack immediate etiological relevance they may nevertheless lead to new insights into the mechanisms of disease progression, with potential benefits in terms of therapy and disease management.

### Data mining

**Figure 5:**
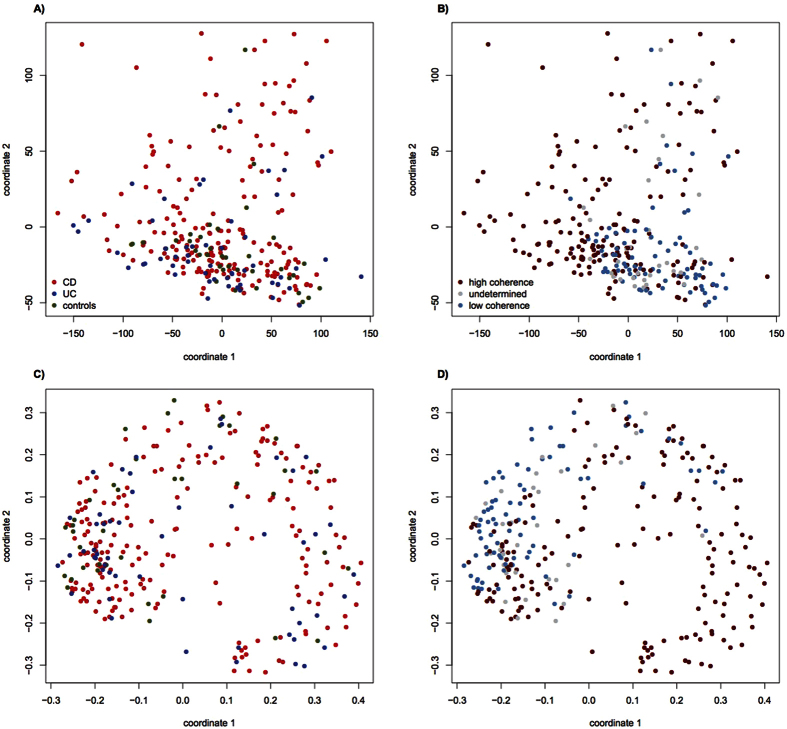
Multidimensional scaling (MDS) analysis of gene expression data (each dot represents an individual sample). (**A**) Euclidean distance, colored according to diagnosis, (**B**) Euclidean distance, colored according to metabolic network coherence, (**C**) binary distance, colored according to diagnosis, (**D**) binary distance, colored according to metabolic network coherence.

Classical data mining aims at discerning patterns in data without invoking additional contextual information. We applied multi-dimensional scaling (MDS) analysis to the original expression profile data of the pediatric IBD patients and controls. When the Euclidean distances between the original DESeq values were subjected to MDS, no particular pattern became apparent (Fig. 5A). However, a different result was obtained when the DESeq values were dichotomized according to whether or not they exceeded ± 3, in which case the respective gene was termed ‘saliently expressed’. Note that ± 3 is an appropriate threshold for z scores like the normalized DESeq values because ± 3 roughly demarcates the 1% quantile of the standard Gaussian distribution. With the dichotomous data, MDS revealed two clusters of expression profiles that could be distinguished well in the first dimension (Fig. 5C,D).

MDS analysis did not reveal any relationship between disease type or case-control status and cluster affiliation (Fig. 5A,C). However, virtually all expression profiles from the low coherence group, assigned to distribution (A) with > 80% certainty, were found to fall into only one of the two binary-distance based MDS clusters. The high coherence group (B) predominated the other cluster (Fig. 5D). Although less well-structured, the Euclidean distance-based MDS plots exhibited a bipartite partition as well (Fig. 5B). Similar results were obtained for IBD patients alone (Supplementary Figure S3).

The fact that MDS of the binary distance data yielded a more clear-cut result than MDS of the original DESeq values may appear surprising at first glance because, from a statistical point of view, dichotomization usually entails a loss of information. However, in the present situation, focusing the analysis upon saliently (i.e., particularly highly or lowly) expressed genes may have been equivalent to highlighting the relevant links between gene activity and metabolism and, at the same time, filtering out the noise that is likely to constitute intermediate expression levels.

**Figure 6:**
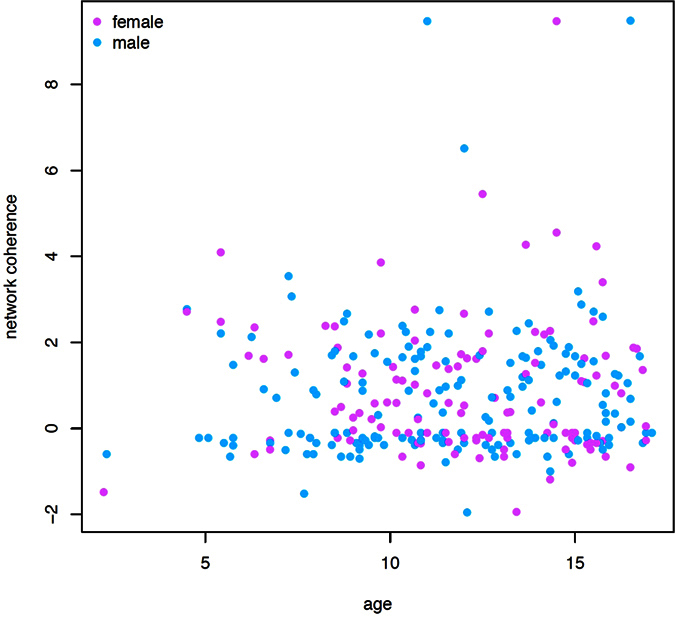
Metabolic network coherence values of all gene expression profiles, arranged by individual age and gender.

In order to assess the possible role of known biological determinants of both gene expression and metabolism, we stratified the distribution of metabolic network coherence values by both age and sex. However, no influence of these two covariates became apparent (Fig. 6).

### Saliently expressed genes

**Figure 7:**
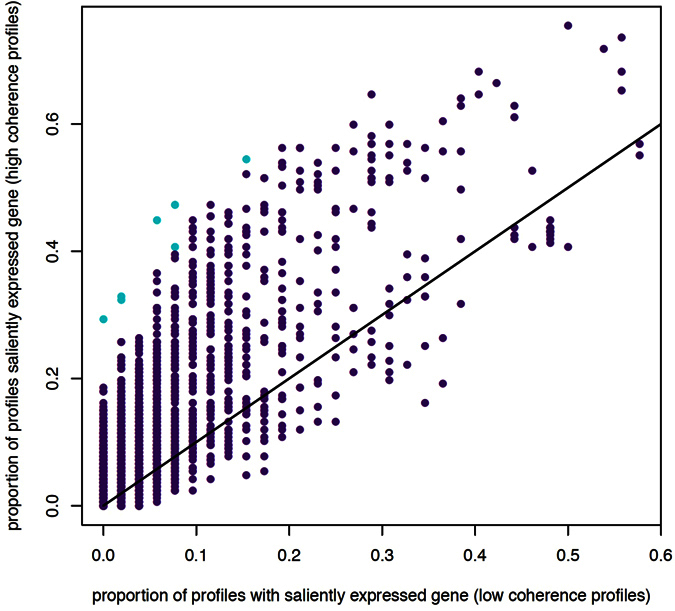
Gene-wise analysis of salient expression in the two coherences groups (each dot represents a gene). Vertical axis: proportion of pediatric IBD patients in the low coherence group for which the respective gene was saliently expressed (i.e. for which the DESeq value exceeded ± 3); horizontal axis: same as vertical axis, but for high coherence group. Genes with statistically significant proportions in the two groups are marked by blue coloring.

For each gene and each coherence group, we determined the proportion of profiles in which the gene was saliently expressed (I,e, DESeq > + 3 or DESeq < −3). When the two proportions were assessed for a statistically significant difference among IBD patients using a Fisher or chi-squared test as appropriate, and allowing for multiple testing, seven genes were found to be saliently expressed more often in one of the two coherence groups (Fig. 7, Table 1).

A change in metabolism has been hypothesized for long to play a role in the etiology of IBD. Early work, focused upon energy homeostasis in intestinal epithelial cells^11^, revealed diminished butyrate oxidation to CO_2_ and ketones as well as a shift to increased glucose and glutamine oxidation in UC patients in a process that potentially compensates for the concurrent decrease in fatty acid oxidation. The importance of fatty acid metabolism in IBD was further highlighted by the observation that the expression of fatty acid synthase and long chain acyl-CoA synthetases (ACSL) 1 and 4 genes is altered in IBD patients, and that this change probably reflects impaired sensing of bile acids via the LXR receptor^28^. Intriguingly, we found two UDP glucuronosyltransferase genes to be saliently expressed more often in the high than the low coherence group of pediatric IBD patients (Table 1). For decades, the UDP glucuronosyltransferases of the intestinal mucosa have been known to contribute to the extrahepatic metabolism of bile acids^29^,^30^, even though the precise role of this process in inflammatory responses is still poorly understood.

IL6 is a cytokine, known to promote intestinal inflammation, that has a clear role in fatty acid metabolism, for example, by stimulating apolipoprotein (a) expression and lipoprotein (a) synthesis in hepatocytes^31^. Along the same vein, the *TM4SF4* gene encodes a transmembrane protein that stimulates thiamine resorption in intestinal epithelial cells^32^. Thiamine, in turn, is an essential component of several co-enzyme complexes, including pyruvate dehydrogenase that catalyzes the formation of Acetyl CoA as a first step in fatty acid synthesis. Interestingly, a variant in *TM4SF4* was recently found to increase the risk for gallstone formation^33^, a disease that involves impaired enterohepatic circulation of bile acids.

In summary, we may surmise that a functional link exists between fatty acid metabolism and inflammation that partly explains why high metabolic network coherence was more prevalent in IBD patients than controls in our study.

## Conclusions

The pronounced heterogeneity of disease progression and therapy response observed among patients with inflammatory bowel diseases (IBD) calls for a more refined classification of cases to benefit both medical research and clinical care^34^. Therefore, a careful assessment of the functional state of patient tissues as captured by high-throughput molecular data appears well warranted.

We used a network approach to analyze gene expression data from pediatric IBD patients and controls, not only to resolve otherwise indiscernible patterns in these data, but also to improve our understanding of the underlying disease mechanisms. The latter was facilitated by our drawing upon more general insights into a particular type of biological system, namely metabolic networks. Two distinct subgroups of expression profiles were identified on the basis of these considerations: one where the metabolic network coherence was high on average and varied substantially between individuals, and one where metabolic network coherence was distinctly lower and less variable. Whilst the latter group dominated the control group, the former was most prevalent in IBD patients. Whether this discrepancy reflects causes or consequences of disease manifestation remains unclear but warrants further exploration.

The metabolic network coherence-based classification of transcriptome profiles showcased here also bears potential for translation into clinical practice in that it opens an additional perspective for the biology-driven stratification of IBD patients. Since the success prospects of pharmacological therapies in IBD or in any other inflammatory disease are likely to be influenced by the peculiarities of the individual metabolism, metabolic network coherence may represent a suitable biomarker to distinguish between responder and non-responders, or to predict side effects, for certain treatments. In addition, as was evidenced by the different prevalence of high and low coherence in patients and controls, metabolic network coherence may also serve as a diagnostic marker, for example, to allow differentiation between IBD and non-IBD intestinal health problems.

Classical data mining was capable of identifying substructure in the gene expression data as well that mirrored the results of the metabolic network coherence analysis. The fact that the two coherence groups could be discerned without invoking the metabolic network itself suggests that the differences between the two patient groups reside at a more comprehensively systemic level, and that metabolism only served as a marker for these differences.

We employed publicly available transcriptome data from intestinal biopsies of mostly therapy-naive pediatric IBD patients. Even though some of the clinical characteristics (no previous immunosuppressive medication, sampling close to first diagnosis, narrow age range) render this group ideal for metabolism-centered analyses, it must be emphasized that pediatric IBD differs from adult IBD in several ways^35^. Moreover, the controls employed in our study were considered “non-IBD” by the treating physicians, but still presented with intestinal health problems. Therefore, it cannot be excluded that presence of the high metabolic network coherence state in this group reflected particular non-inflammatory factors such as, for example, a specific infection. Therefore, it must be verified explicitly whether metabolic network coherence is also bimodal in adult IBD patients or in adults in general.

The present study also highlighted two synergistic aspects of the combination of network analysis and classical data mining. On the one hand, network analysis provides a means to use external contextual information to facilitate a better understanding of the results of classical data mining. On the other hand, classical data mining can lend statistical support to the qualitative results of network analysis. Nevertheless, experimental studies are now required to link the two distinct states of gene expression inferred by our combined *in silico* approach to etiological pathways. Such linkage would represent yet another critical step towards network medicine fulfilling its ultimate claim, namely to benefit patients by way of clinically actionable results.

## Methods

### Data

**Figure 8:**
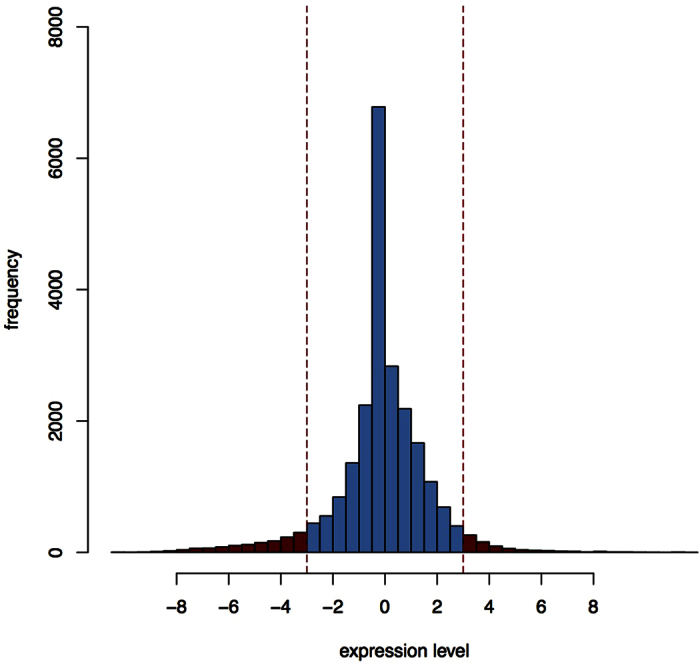
Exemplary distribution of DESeq values in a single gene expression profile. Saliently expressed genes are defined as genes with a DESeq value exceeding ± 3.

In this study, we used RNA-seq data of the RISK cohort^13^ comprising 321 intestinal tissue samples from treatment-naive pediatric patients with a confirmed diagnosis of Crohn disease (CD) or ulcerative colitis (UC), and from age-matched controls. The proband age ranged from 2 to 17 years, 40% of individuals were female. The CD group comprised 218 patients, 61 individuals were diagnosed with UC and 42 were controls. Ileal biopsies were taken from all individuals and gene expression was measured by RNA-seq. The original data were processed further using the DESeq algorithm for RPKM normalization. Recruitment procedure, data quality measures and data processing are described in detail in the original report^13^. Our analyses employed data publicly available at http://www.ncbi.nlm.nih.gov/geo/query/acc.cgi?acc=GSE57945. The original DESeq data consisted of one continuous score per gene (or transcript). Since metabolic network coherence analysis requires a binary score per gene, however, we had to dichotomize the data, labeling genes with a DESeq value < −3 or > + 3 as ‘saliently expressed’ (Fig. 8). The choice of this threshold was motivated by the fact that DESeq values are z scores, and that ± 3 roughly demarcates the 1% and 99% quantile, respectively, of the standard Gaussian distribution.

### Metabolic network coherence

For metabolic network coherence analysis, we mapped the expression profiles of patients onto reaction-centric metabolic networks and studied the ensuing effective metabolic networks (i.e. subnetworks spanned by the saliently expressed genes *e*). For an effective network *G*_*e*_(*V,E*) with a set of vertices (reactions) *V* = {*r*_*1*_*, r*_*2*_*, …, r*_*K*_} and edges *E*, metabolic coherence *C* is computed as follows: Let *k*_*i*_ denote the degree of vertex *r*_*i*_ in the effective network and let *K*_*c*_ be the number of vertices *r*_*i*_ for which *k*_*i*_ > 0. The connectivity of the effective network (i.e., the number of reactions with non-zero degree divided by the size *K* of the effective network, R = *K*_*c*_/*K*) reveals how ‘meaningful’ the gene-gene correlation in different expression state is from a metabolic perspective. An observed ratio R can be tested for statistical significance by means of comparing it to the null distribution. Here, the null distribution was simulated by randomly drawing the same number of saliently expressed metabolic genes from the set of all metabolic genes, leading to a set of ratios {R_1_^(r)^, R_2_^(r)^, … R_N_^(r)^} for random data with mean <*R*^(*r*)^> and standard deviation *σ*(*R*^(*r*)^). The metabolic coherence *C*(*e*) of a gene expression profile *e* is then defined as the z-score with respect to the null distribution, i.e. C(e) = (R-<*R*^(*r*)^>)/*σ*(*R*^(*r*)^). In cases, where the effective network comprised less than two nodes (19 CD, 7 UC, 4 controls), no metabolic coherence value could sensibly be computed.

### Statistical analysis

The distribution of metabolic network coherence in different sub-groups was subjected to mixture analysis as implemented in SAS procedure FMM (version 9.5; SAS Institute Inc., Cary, NC, USA). In each case, the best fit was observed for two Gaussian distribution, albeit mixed at different proportions. Then, the posterior probability of being sampled from one of the two distributions was calculated of each individual profile. If one of the two posterior probabilities exceeded 0.8, the profile was classified as ‘highly’ or ‘lowly’ coherent, depending upon the respective distribution; otherwise, the profile was classified as ‘undetermined’ (Table 2). Differences between the metabolic network coherence distributions in different groups of profiles were assessed for statistical significance using a Kruskal-Wallis as implemented in SAS procedure NPAR1WAY.

### Data mining

Multidimensional scaling (MDS) analysis was performed with R v.3.1.3^36^. As continuous input, we used Euclidean distances between gene-specific DESeq values. In addition, binary distances between dichotomized expression levels were calculated as implemented in R-command *mds*.

### Graphs

Metabolic gene networks were generated from Recon 2 v.3 by connecting any two genes that shared a gene-enzyme-reaction-enzyme-gene relationship while excluding metabolites belonging to a list of ‘currency metabolites’ (e.g., ATP, H_2_O). Currency metabolites were eliminated by removing the top 5% of metabolites after sorting them by their node degree in the gene-centric metabolic network. This way, 1009 of the 1101 original nodes remained in the network.

## Additional Information

**How to cite this article**: Knecht, C. *et al.* Distinct metabolic network states manifest in the gene expression profiles of pediatric inflammatory bowel disease patients and controls. *Sci. Rep.* **6**, 32584; doi: 10.1038/srep32584 (2016).

## Supplementary Material

Supplementary Information

## Figures and Tables

**Table 1:** Genes expressed saliently at significantly different proportions in low and high coherence expression profiles (IBD patients only).

Gene	Gene product	P value	Number of expression profiles	
			low coherence (n = 52)	high coherence (n = 167)
*UGT1A8*	UDP glucuronosyltransferase 1 family, polypeptide A8	4.8 × 10^−7^	0	49
*UGT1A6*	UDP glucuronosyltransferase 1 family, polypeptide A6	3.0 × 10^−6^	4	68
*TM4SF4*	transmembrane 4 L six family member 4	8.0 × 10^−7^	1	55
*IL6*	interleukin 6	8.4 × 10^−7^	1	54
*C9orf71 (TMEM252*)	transmembrane protein 252	1.7 × 10^−6^	8	91
*LAMC3*	laminin subunit gamma 3	3.8 × 10^−8^	3	75
*TFAP2C*	transcription factor AP-2 gamma	6.2 × 10^−8^	4	79

P values refer to a Fisher or chi-squared test, as appropriate, comparing the number of profiles in which a given gene was saliently expressed between the low and high coherence group.

**Table 2:** Number of high and low coherence expression profiles in different phenotypic subgroups.

Diagnosis	High coherence	Low coherence	Unclassified
CD	132	43	24
UC	35	9	10
IBD (UC + CD)	167	52	34
Controls	9	28	1
