# Supplementary material for: Distinct metabolic network states manifest in the gene expression profiles of pediatric inflammatory bowel disease patients and controls
Source: Sci Rep. 2016 Sep 2;6:32584. doi: 10.1038/srep32584 (PMC5009330; doi:10.1038/srep32584)
Supplement: Supplementary Information [file srep32584-s1.pdf]

Supplementary Material

Distinct metabolism-related states manifest in the gene expression profiles of pediatric inflammatory bowel disease patients and controls

Carolin Knecht, Christoph Fretter, Philip Rosenstiel, Michael Krawczak, Marc-Thorsten Hütt

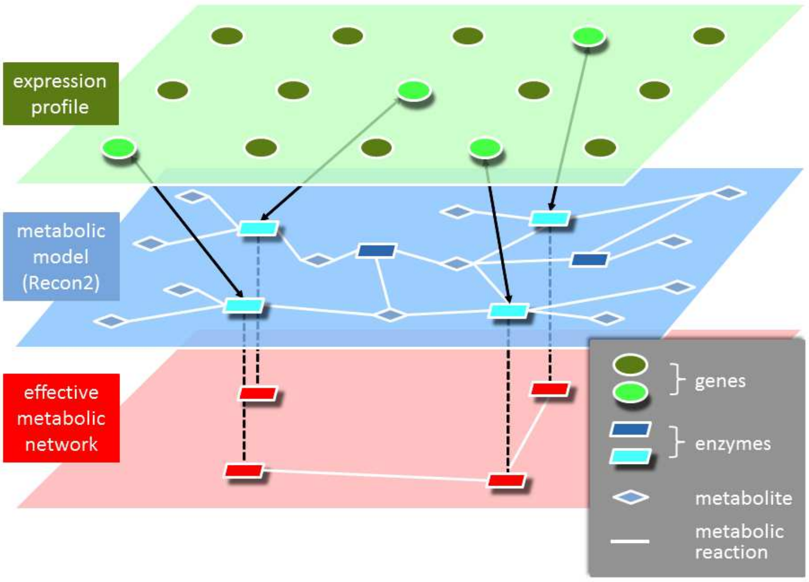

Legend to Supplementary Figure S1

Schematic representation of metabolic network coherence analysis. Each expression profile is converted into a vector comprising one binary signal per gene, indicating whether the gene is highlighted (i.e. 'saliently expressed') or not. Then, the binary signals are mapped onto the Recon 2 metabolic network according to the functional correspondence between genes and enzymes. This mapping generates an 'effective' metabolic network, i.e. a set of highlighted enzymes (or genes) indirectly connected, or not, by metabolic reactions. Finally, the ratio of connected to total highlighted enzymes is compared to equivalent ratios obtained from sets of randomly highlighted genes so as to yield the metabolic network coherence of the expression profile (see Methods for details on the final step).

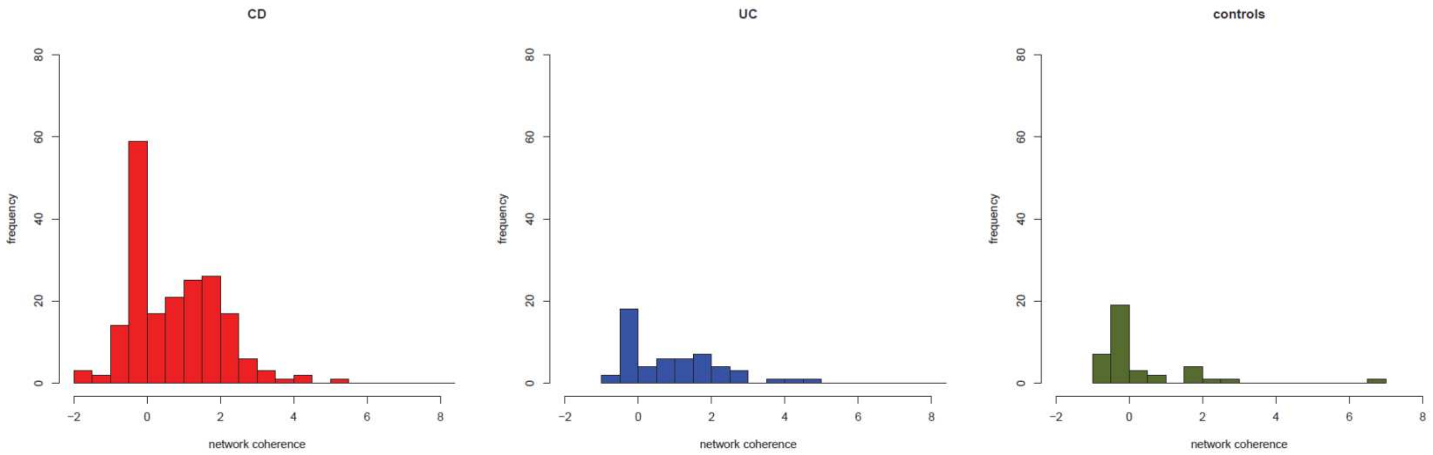

Legend to Supplementary Figure S2

Distribution of metabolic network coherence in intestinal samples from different phenotypic subgroups.

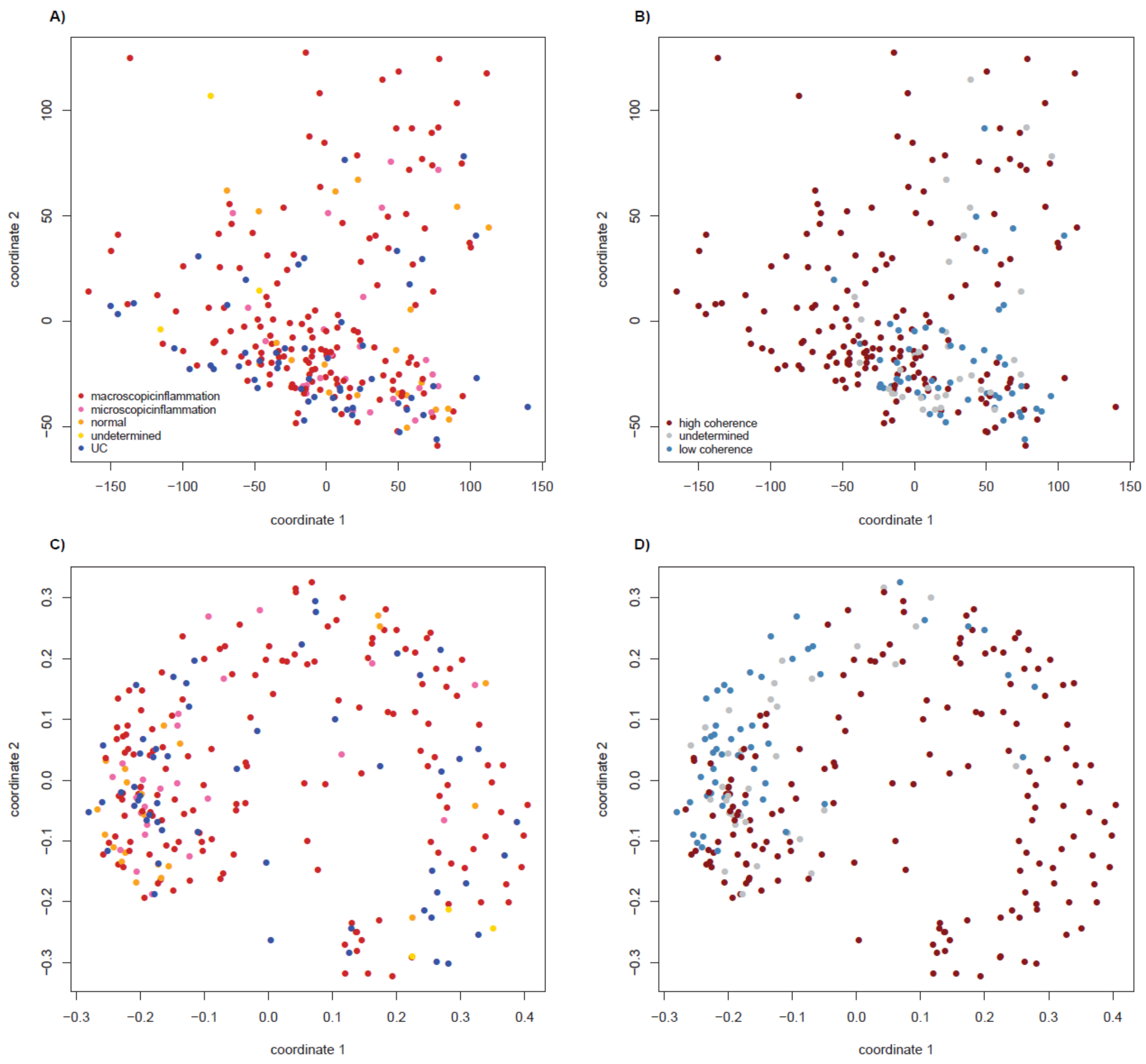

Legend to Supplementary Figure S3

Multidimensional scaling (MDS) analysis of gene expression data of IBD patients only (each dot represents an individual sample). (A) Euclidean distance, colored according to histopathology, (B) Euclidean distance, colored according to network coherence, (C) binary distance, colored according to histopathology, (D) binary distance, colored according to network coherence.

Supplementary Table S1

	Distribution	Mean	Variance	Mixing Probability
Crohn disease	A	-0.294	0.221	0.481
	B	1.525	0.563	0.519
Ulcerative colitis	A	-0.208	0.025	0.348
	B	1.339	0.969	0.652
